# Paradoxical Reactions of Central Nervous System Tuberculosis: Report of Three Immunocompetent Cases

**DOI:** 10.1155/crdi/5416948

**Published:** 2025-08-21

**Authors:** Eduardo Mariño, Jorge Rodríguez-Pardo, Laura Vidal, Gerardo Zmork, Alicia Garcial-Leal, Beatriz Díaz-Pollán, Laura Lacruz

**Affiliations:** ^1^Neurology Department and Stroke Center, Hospital La Paz Institute for Health Research–IdiPAZ, La Paz University Hospital–Universidad Autónoma de Madrid, Madrid, Spain; ^2^Infectious Diseases Unit, Internal Medicine Department, IdiPAZ (La Paz Institute for Health Research), La Paz University Hospital, Madrid, Spain; ^3^CIBERINFEC (Centro de Investigación Biomédica en Red de Enfermedades Infecciosas), Instituto de Salud Carlos III, Madrid, Spain

**Keywords:** case report, central nervous system tuberculosis, immunocompetent patients, paradoxical reactions, tuberculosis therapy

## Abstract

**Introduction:** Paradoxical reactions during tuberculosis (TBC) therapy are characterized by clinical or radiological worsening of preexisting tuberculous lesions or the appearance of new manifestations following appropriate TBC treatment. Identifying this phenomenon is crucial, since it can be mistaken with treatment failure or relapse. Although widely described in HIV patients following immune reconstitution inflammatory syndrome, the literature on HIV-negative patients is scarce.

**Case Series:** We present three cases of immunocompetent patients with central nervous system tuberculosis (CNS-TBC) who developed paradoxical reactions following appropriate TBC therapy. These included diverse clinical and radiological manifestations, such as persistent headaches, apparition or progression of tuberculomas, cerebral infarcts, and dorsal myelitis. Paradoxical reactions occurred within an average of 2.5 months from the start of anti-TBC treatment.

**Conclusion:** Our findings underscore the importance of closely monitoring patients following anti-TBC treatment to identify potential complications rapidly. Paradoxical reactions due to exaggerated immune response to *Mycobacterium tuberculosis* complex antigens should be considered in a thorough differential diagnosis including other CNS infections, granulomatous or neoplastic disorders, treatment failure, or treatment-related toxicities. Ensuring adequate adherence to anti-TBC treatment and immunosuppressants is essential in such cases.

## 1. Background

A paradoxical reaction to tuberculosis (TBC) therapy is defined as the clinical or radiological worsening of preexisting tuberculous lesions or the appearance of new ones in patients after receiving appropriate anti-TBC treatment. It most often emerges within the first 4–8 weeks, although cases have been reported as early as the second week of therapy [1]. Its diagnosis requires to rule out other conditions such as treatment failure, multidrug resistance, drug toxicity, or clinical deterioration due to the coexistence of another central nervous system (CNS) infection) [2].

A paradoxical reaction to TBC therapy is considered a manifestation of TBC-associated immune reconstitution inflammatory syndrome (IRIS). Two forms of TB-IRIS are recognized: paradoxical IRIS, where there is clinical or radiological deterioration despite appropriate treatment, and unmasking IRIS, where previously undiagnosed TB presents with an exaggerated inflammatory response after immune recovery [3]. The current knowledge of paradoxical reaction points to an exaggerated immune response to *Mycobacterium tuberculosis* complex antigens as the underlying cause in patients on effective anti-TBC treatment [4]. In human immunodeficiency virus (HIV) coinfected patients, a paradoxical manifestation has been well described over the years, especially about the beginning of antiretroviral and anti-TBC treatment in patients with high viral load and low CD4+ cell counts [5]. However, current knowledge of HIV-negative patients is limited to small series and reports [6]. In previous studies, the frequency of CNS paradoxical manifestations among HIV-negative patients with tuberculous meningitis (TBM) varied from 7% to 56% [1], and its mortality from 9% to 35% [7]. CNS symptoms may include headache, mental confusion, seizures, cranial nerve palsy, and focal deficits of the limbs, secondary to different complications including the expansion of preexisting cerebral tuberculoma or the appearance of new ones, hydrocephalus, optochiasmatic and spinal arachnoiditis, cerebral vasculitis, and spinal cord involvement, among others [8].

We present three cases of HIV-negative patients with central nervous system tuberculosis (CNS-TBC) who developed paradoxical reactions after treatment. To date, there are no clinical trials or controlled prospective studies that establish the most appropriate management for this condition that could be potentially fatal. We aim to raise awareness of paradoxical reactions and the importance of closely monitoring CNS-TBC patients after treatment initiation.

## 2. Case Presentation

A summary of the characteristics of the three cases in this series is provided in Table S1.

### 2.1. Case 1

A 37-year-old male was diagnosed with disseminated TBC involving the joints, bones, lungs, lymph nodes, and brain, with *Mycobacterium bovis* isolated from joint fluid. His medical history includes a daily consumption of 10 cigarettes and heart failure with a left ventricular ejection fraction (LVEF) of 35%, resulting from a previous cardiac stab injury. No identifiable zoonotic-TB risk factors were present: the patient denied consumption of unpasteurized dairy products, contact with livestock or wildlife, and travel to endemic areas.

**Figure 1:**
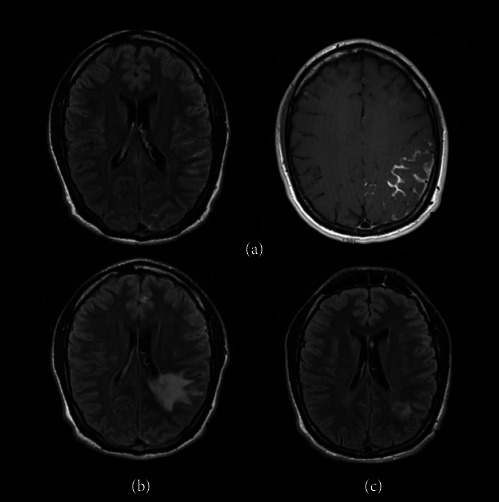
Evolution of CNS lesions in MRI. (a) Pretreatment: thickened left frontal, parietal, occipital cortex and leptomeningeal enhancement. (b) Post 3 month treatment: increased thickness and density in left parietal white matter, indicating cerebritis and edema. (c) Post 1 month corticosteroid reintroduction: overall improvement, residual left parietal white matter edema.

Initially, the patient presented with fever, headache, mild weakness of the right limbs, and acute monoarthritis of the right foot. Cranial MRI showed lesions in the left middle cerebellar peduncle and left midbrain (compatible with tuberculomas), and thickening of the left insular, frontal, parietal, and occipital cortex, with a notable leptomeningeal enhancement (Figure 1(a)). Cerebrospinal fluid (CSF) analysis was unremarkable (Table S1). Quantiferon test was found positive. Body CT showed cervical, hilum, mediastinum, and peritoneal lymphadenopathy, a micronodular lung pattern, and splenomegaly. Acid-fast bacilli were observed in smear microscopy only in right foot joint fluid, with subsequent isolation of *Mycobacterium bovis*, and the nucleic acid amplification test (NAAT) (Xpert MTB/RIF assay) showed no resistance to rifampicin. No microorganisms were observed or isolated in the remaining samples (CSF, sputum, and bronchial aspirate).

Treatment was initiated with the fixed-dose combination Rimstar (each tablet containing rifampicin 150 mg, isoniazid 75 mg, pyrazinamide 400 mg, and ethambutol 275 mg). The patient, weighing 60 kg, received 4 tablets once daily, providing a total daily dose of rifampicin 600 mg, isoniazid 300 mg, pyrazinamide 1600 mg, and ethambutol 1100 mg. Adjunctive therapy with dexamethasone 4 mg every 8 h was started, with gradual tapering over 2 months. During this period, the patient showed progressive clinical improvement. After 2 months, the treatment regimen was reduced to rifampicin and isoniazid (HR). Subsequently, 3 months later, the patient presented to our clinic with worsening of the hemiparesis. A follow-up MRI revealed increased leptomeningeal enhancement in the left temporal, parietal, and occipital regions, along with growth of previous tuberculomas and associated vasogenic edema compared to the previous MRI (Figure 1(b)). After ruling out other CNS infections, dexamethasone was increased to 4 mg every 8 h. Clinical and radiological improvement was confirmed after 1 month (Figure 1(c)).

### 2.2. Case 2

**Figure 2:**
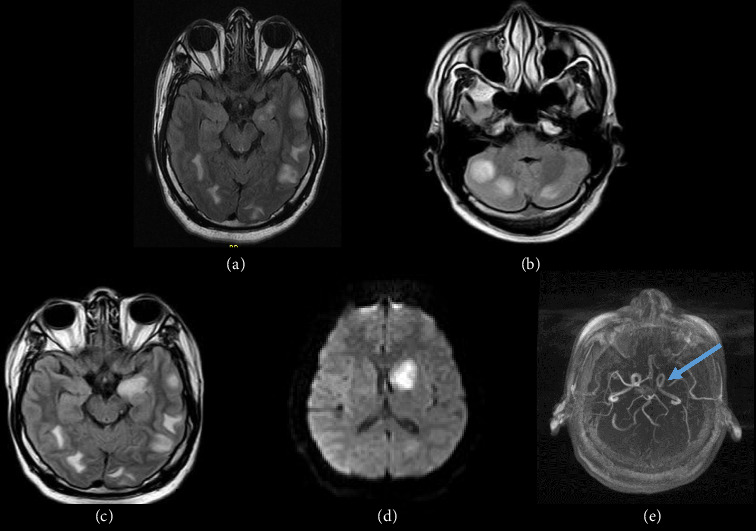
Brain MRI/MRA findings in a CNS tuberculosis patient with paradoxical response. (a, b) Baseline FLAIR: multiple supra- and infratentorial cortico-subcortical tuberculomas. (c) Post 2 month treatment: known tuberculomas with increased left basal ganglia edema. (d) DWI at 2 months: new ischemic lesion in left basal ganglia. (e) MRA: stenosis of left M1 and A1 segments (arrow).

A 29-year-old man with no previous relevant history presented to the emergency department with fever (39°C), headache, and vomiting. A lumbar puncture, a chest CT, and a brain MRI were performed. A NAAT for *M. tuberculosis* complex was negative in CSF but positive in bronchoalveolar lavage. Brain MRI showed several cortico-subcortical supra and infratentorial tuberculomas (Figures 2(a) and 2(b)). Treatment was initiated with 5 Rimstar tablets once daily (the patient weighed around 80 kg), providing a total daily dose of rifampicin 750 mg, isoniazid 375 mg, pyrazinamide 2000 mg, and ethambutol 1375 mg. Adjunctive dexamethasone 4 mg every 8 h was initiated for 2 months, with clinical improvement.

Two months later, the patient was admitted with severe headache, hemiparesis, and right facial palsy. MR-angiography (MRA) showed an increase in the number of previously mentioned tuberculomas and a lesion of recent ischemia in the territory of the left middle cerebral artery (MCA), together with contrast-enhancing stenosis of the left MCA (M1 segment) and left anterior cerebral artery (A1 segment) (Figures 2(c), 2(d), and 2(e)). Therapy with HZRE was resumed along with 400 mg of oral moxifloxacin for 2 months, 100 mg of acetylsalicylic acid, and dexamethasone 0.4 mg/kg with gradual tapering. The patient showed both clinical and radiographic improvement in the following 6 months when corticosteroids and acetylsalicylic acid were stopped. After 10 months, rifampicin and isoniazid were also interrupted. The patient showed full recovery and control MRA revealed no new lesions and resolution of the stenosis.

### 2.3. Case 3

A 45-year-old man without relevant history was admitted to our emergency department with cough, fever, headache, and lumbar pain. The patient reported a weight loss of 13 kg in the last 4 months. Body CT was noteworthy for a pulmonary micronodular pattern and multiple retroperitoneal adenopathies. Brain MRI revealed multiple small solid nodular focal lesions with parenchymal enhancement located in the left corona radiata, right insula, superior cerebellar peduncle, and right cerebellar hemisphere. These lesions showed no mass effect or significant perilesional edema and were consistent with tuberculomas. Urine cultures and smears were negative. NAAT for *Mycobacterium tuberculosis* complex in CSF was positive. A diagnosis of miliary TBC with CNS involvement was made. Treatment was initiated with 4 Rimstar tablets once daily (the patient weighed 65 kg), providing a total daily dose of rifampicin 600 mg, isoniazid 300 mg, pyrazinamide 1600 mg, and ethambutol 1100 mg. Adjunctive dexamethasone 4 mg every 8 h was initiated, with partial improvement. *Mycobacterium tuberculosis* was isolated in culture after 26 days of growth.

**Figure 3:**
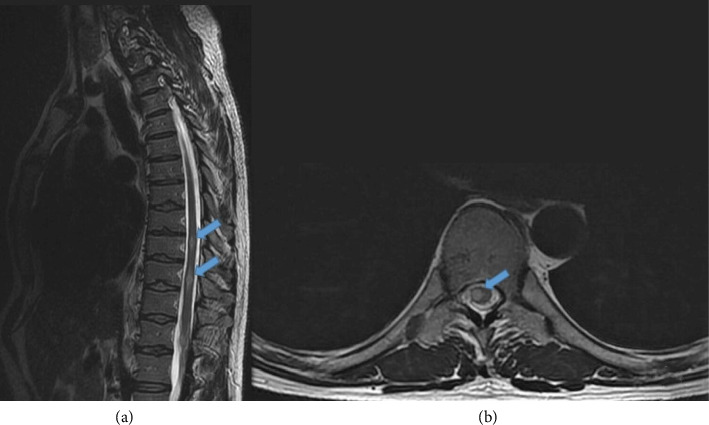
MRI of the dorsal spine with T2 (a, b) sequences. High-signal T2 plaques of variable size between 5 and 12 mm are observed, located in patchy form along the entire dorsal spine, compatible with myelitis in relation to a paradoxical reaction to tuberculosis treatment.

After 2 months of treatment, the patient complained of paresthesias in the distal region of the four limbs together with lumbar pain irradiated to lower limbs. A spinal MRI revealed spinal signal changes with contrast enhancement along the dorsal medulla compatible with myelitis (Figure 3). Brain MRI revealed two new tuberculomas. Suspecting a paradoxical reaction to the treatment, the HZRE scheme and prednisone 30 mg for 4 months were restarted.

Three months after the paradoxical reaction, the treatment was switched to HR. At 6 months of follow-up, the patient showed significant clinical improvement, with only mild paresthesia remaining in the right lower limb. Brain MRI revealed stable lesions with no signs of progression, while the full spine MRI demonstrated significant radiological improvement. After 10 months of treatment, and due to the positive clinical outcome, the TBC treatment was discontinued.

## 3. Discussion

This work illustrates the clinical complexity of the diagnosis and management of CNS-TBC and paradoxical reactions to the treatment. Paradoxical reactions have been widely described in HIV-positive patients following the availability of effective antimicrobial treatment for both HIV and TBC [9]. However, the evidence on immunocompetent patients is scarce, and most studies have been conducted in countries with a high prevalence of TBC. We present a case series of paradoxical reactions to CNS-TBC in a western country to underscore the importance of considering this condition, whose severity can range from asymptomatic to fatal [2].

In cases with strong clinical suspicion of CNS-TBC, first-line tuberculostatics (HZRE) and adjuvant corticosteroids should be started before microbiological confirmation. The treatment typically extends over a period of 2 months, followed by a 7–9-month regimen with HR [10]. While we followed the WHO-endorsed RHZE, it is worth noting that our cases were diagnosed before newer recommendations—such as those in the 2024 Red Book—advocated replacing ethambutol with a fluoroquinolone or protionamide/ethionamide for better CNS penetration [11]. Corticosteroids have shown to decrease short-term mortality, but they have not shown benefits for long-term disability [12]. After tuberculostatic and corticosteroid treatment, all of our patients showed an initial improvement with later clinical worsening in the form of paradoxical reactions, with different clinical and radiological manifestations. Two patients of this series had new focal neurological deficits (Cases 1 and 2), and two individuals experienced ongoing headache (Cases 2 and 3). Cases 2 and 3 showed supra- and infratentorial lesions at MRI that were consistent with tuberculomas, along with new lesions or progression of prior ones. One patient exhibited worsening focal leptomeningeal enhancement, one had cerebral infarct, and another one had dorsal myelitis. Mean time from treatment initiation and paradoxical reactions was 2.5 months, similar to that shown in different studies [1].

Cerebral prior indetectable tuberculomas are the most frequently reported manifestation of paradoxical reactions in HIV-negative patients [8]. Cerebral tuberculomas are conglomerated caseous foci containing mycobacteria in a latent state. The mechanism by which the reaction occurs is thought to be the result of an exaggerated immune response to *Mycobacterium* antigens in patients on effective anti-TBC treatment rather than due to progressive uncontrolled mycobacterial replication. These cell wall antigens may stimulate an exaggerated inflammatory reaction in the host [13]. Interestingly, Case 1 in our series was caused by *Mycobacterium bovis*, an agent typically associated with extrapulmonary or disseminated TB [14]. Since disseminated disease has been linked to a higher incidence of paradoxical reactions [15], one could hypothesize that *M. bovis* infection—through its propensity for widespread disease—might contribute to this risk. Further studies are needed to explore this potential association.

Increased exudates in the basal cisterns are the second most frequent finding in paradoxical reactions. They can also be present in the interpeduncular cisterns, around the optic chiasm, in the suprasellar cisterns and ambiens, and in the cisterns of Sylvius, as described in our series [16]. Moreover, basal exudates produce inflammatory changes in the vessels, predominantly those of the circle of Willis. Vascular complications frequently occur paradoxically and manifest as stroke [17]. These infarcts mainly involved the lateral lenticulostriate arteries (73%), medial lenticulostriate arteries (41%), and perforators from posterior cerebral artery (38%) and are located within the so-called “*TBC zone*,” which includes the periventricular regions, internal capsule, thalamus, and basal nuclei [18]. Case 2 exemplifies this manifestation, showing ischemic lesions in the left basal nuclei. Involvement of the spinal cord has also been described. This includes radiculomyelitis (Case 3), spinal tuberculoma, syringomyelia, vertebral TBC, and, very rarely, spinal tuberculous abscess [8].

Noteworthily, in our series, *Mycobacterium tuberculosis* complex infection was confirmed using Xpert MTB/RIF in joint fluid, bronchoalveolar lavage, and CSF in Cases 1, 2, and 3, respectively. According to the latest WHO recommendations, in adults presenting with signs and symptoms of TB meningitis, Xpert MTB/RIF should be used as the initial diagnostic test for CSF [19]. Additionally, this test may also be employed as the initial diagnostic tool for synovial fluid [19]. The Xpert MTB/RIF assay has emerged as a transformative tool in TB diagnostics, offering rapid detection of *Mycobacterium tuberculosis* and rifampicin resistance with high specificity (> 98%) across various sample types. Its sensitivity, however, varies considerably depending on the biological specimen. For CSF, individual prospective studies using smaller volumes or unprocessed samples report Xpert MTB/RIF sensitivities ranging from 20% to 60% and Xpert MTB/RIF Ultra sensitivities of 44%–77%. Even with Xpert Ultra, a negative result does not reliably exclude TB meningitis when clinical suspicion is high [20]. Sensitivity can be further improved by collecting larger CSF volumes (≥ 6–10 mL) and concentrating the specimen by high-speed centrifugation (3000 × g for 15–20 min), as recommended by WHO and expert guidelines [10]. Meta-analyses that include studies with optimized CSF processing report pooled Xpert sensitivities of around 80% against culture [21]. These findings underscore the continued importance of culture as the gold standard, particularly for confirmatory testing and comprehensive diagnostic accuracy [22].

After clinical or radiological worsening, other CNS infections, granulomatous, or neoplastic disorders were investigated and excluded. Furthermore, we consistently monitored for adherence to the therapeutic regimen, actively investigated for any treatment-related toxicities, and evaluated the potential of treatment failure due to microbiological resistance [23]. Using the GeneXpert technique, sensitivity to rifampicin was confirmed in all of our patients. In addition, serial brain MRI can help distinguish a favorable response to immunomodulatory therapy from ongoing active disease: in successful cases, paradoxical lesions typically show a gradual loss of T2/FLAIR hyperintensity over time, whereas persistent or increasing hyperintensity suggests treatment failure or progression [24].

For paradoxical manifestations, there are no consensus on surveillance, diagnosis, and treatment. Case reports and case series have mentioned the use of different doses of corticosteroids, mostly according to the Thwaites protocol, designed for TBM [25]. Although dose regime and duration of corticoid treatment have not been stablished, it is advisable to maintain corticoid therapy for at least 3 months. Other immunomodulatory treatments such as anti-TNF-α, adalimumab, thalidomide, or cyclophosphamide have also been tested [1]. It is important to highlight that, in the context of TBM, adjunctive aspirin therapy has been shown to reduce the incidence of new-onset stroke (NNT = 10) when compared to placebo [26], and this approach is also recommended as adjunctive therapy in the 2024 *Sanford Guide to Antimicrobial Therapy* [27]. However, there is still a lack of consensus regarding its routine use, which has led to the initiation of a Phase III randomized controlled trial (INTENSE-TBM) [28] that aims to provide further insight into this issue.

Due to the positive response to corticoid therapy, no additional treatments were considered in our series. In patients with CNS-TBC paradoxical reactions, up to 35% mortality has been reported [7]. Hence, early identification and treatment can be crucial to improve clinical outcomes in this setting.

This study has limitations inherent to its nature of case series. Our findings do not allow definite recommendations for the monitoring, diagnosis, and management of CNS-TBC patients. However, we believe it contributes to increasing knowledge of the condition and could encourage future studies to be conducted.

## 4. Conclusions

CNS-TBC treatment paradoxical reactions can have a variable presentation, also in immunocompetent patients. Close monitoring of patients for early identification and treatment could help to avoid serious complications. Prospective studies are needed to establish diagnostic and treatment protocols.

## Data Availability

The data that support the findings of this study are available on request from the corresponding author. The data are not publicly available due to privacy or ethical restrictions.

## Supporting Information

Table S1. Summary of clinical presentation, laboratory findings, neuroimaging features, microbiological results, treatment regimens (including weight-based dosing), and outcomes of the three reported cases of CNS tuberculosis with paradoxical reactions.
