# Supplementary material for: Paradoxical Reactions of Central Nervous System Tuberculosis: Report of Three Immunocompetent Cases
Source: Case Rep Infect Dis. 2025 Aug 21;2025:5416948. doi: 10.1155/crdi/5416948 (PMC12393930; doi:10.1155/crdi/5416948)
Supplement: Supporting Information — Additional supporting information can be found online in the Supporting Information section. [file 5416948.f1.docx]

|  | **CASE 1** | **CASE 2** | **CASE 3** |
| --- | --- | --- | --- |
| **Sex** | Man | Man | Man |
| **Age, years** | 38 | 29 | 45 |
| **Presenting symptoms** | Left ankle pain and right hemiparesis | General malaise, fever and headache | General malaise, dry cough and fever |
| **CSF initial findings** |  |  |  |
| Glucose, mg/dL | 53 (serum 110) | 32 (serum 110) | 39 (serum 139) |
| Proteins, mg/dL | 67.8 | 106.2 | 308 |
| Nucleated cells, n/mm^3^ | 5 | 145, lymphocytes 87% | 100, lymphocytes 90% |
| ADA, IU/L | Negative | 12 | 12 |
| **Initial findings on brain MRI** | Left parietotemporal leptomeningeal infiltrate | Supra- and infratentorial tuberculomas | Supra- and infratentorial tuberculomas |
| **Other organs affected** | Joint, bone, pulmonary, lymphatic | Pulmonary and lymphatic | Pulmonary and lymphatic |
| **Initial CNS-TBC treatment** | HZRE 2 months, following HR 7 months | HZRE 2 months, following HR 10 months | HZRE 2 months, following HR 8 months |
| **Initial adjuvant treatment** | Dexamethasone 4mg/8h 2 months | Dexamethasone 4mg/8h 2 months | Dexamethasone 4mg/8h 2 months |
| **Time to onset of paradoxical reaction, months** | 3 | 2 | 2 |
| **Types of paradoxical reactions** | Increased leptomeningeal infiltrate | Increase and new tuberculomas Cerebral infarction | New cerebral tuberculomas Myelitis |
| **Paradoxical reaction treatment** | Dexamethasone 4mg every 8 hours for 2 months, with a gradual tappeting throughout this period | Initially, dexamethasone was prescribed at a dose of 0.4 mg/kg for six months, with a gradual reduction throughout this period | Initially Prednisone 30mg every day for 4 months, with a gradual tappeting throughout this period |
|  |  | HZRE and Moxifloxacin 2 months, then HR 8 months left (total: 12 months) | HZRE 3 months, then HR  5 months left (total: 10 months) |
|  |  | Aspirin 100mg for 6 months |  |
| **Functional outcomes at 6 months** | Asymptomatic | Asymptomatic | Persistent low back pain |
